# Efficacy of a Multistrain Synbiotic Treatment in Acute and Post-Acute COVID-19 Patients: A Double-Blind, Placebo-Controlled Randomized Trial

**DOI:** 10.3390/microorganisms12071443

**Published:** 2024-07-16

**Authors:** Maria Letizia Giancola, Andrea Fontana, Concetta Panebianco, Antonio Mazzarelli, Alessia Beccacece, Patrizia De Marco, Giovanna Cocomazzi, Chiara De Giuli, Germana Grassi, Carla Fontana, Giorgio Maria Baldini, Viviana Contu, Massimiliano Copetti, Francesco Perri, Emanuele Nicastri, Valerio Pazienza

**Affiliations:** 1 National Institute for Infectious Diseases, INMI “Lazzaro Spallanzani”, IRCCS, 00149 Rome, Italy; mletizia.giancola@inmi.it (M.L.G.); antonio.mazzarelli@inmi.it (A.M.); beccacece.alessia@gmail.com (A.B.); patrizia.demarco@inmi.it (P.D.M.); chiara.degiuli@inmi.it (C.D.G.); germana.grassi@inmi.it (G.G.); carla.fontana@inmi.it (C.F.); 2 Biostatistic Unit, Fondazione-IRCCS “Casa Sollievo della Sofferenza” Hospital, 71013 San Giovanni Rotondo, FG, Italy; a.fontana@operapadrepio.it (A.F.); m.copetti@operapadrepio.it (M.C.); 3 Gastroenterology Unit, Fondazione-IRCCS “Casa Sollievo della Sofferenza” Hospital, Opera di San Pio da Pietrelcina, 71013 San Giovanni Rotondo, FG, Italy; panebianco.c@gmail.com (C.P.); g.cocomazzi@operapadrepio.it (G.C.);; 4 AO Consorziale Policlinico di Bari, Università Aldo Moro di Bari, 70121 Bari, BA, Italy; gbaldini97@gmail.com; 5 Integrative Medicine Unit, Humanitas Gradenigo, Corso Regina Margherita 8/10, 10153 Torino, TO, Italy; viviana.contu@gradenigo.it

**Keywords:** SARS-CoV-2, microbiota, probiotics

## Abstract

Background and Aims: Several studies reported the effect of COVID-19 on inducing gut dysbiosis, which is also correlated with disease severity. This study aims to investigate the effect of a nutraceutical formula on the shift of microbiota profiles and, secondly, on the clinical–pathological parameters of acute and post-acute COVID-19 patients. Methods: In this randomised, double-blind, placebo-controlled trial conducted at National Institute for Infectious diseases (INMI) Lazzaro Spallanzani (Italy), 52 patients were randomly assigned (1:1) to receive a multistrain synbiotic formula (Kebirah^®^) or placebo orally for 35 days at COVID-19 diagnosis. Health professionals, investigators, and patients were masked to group assignments. The V3–V4 hypervariable region of 16S rRNA gene sequencing was employed to study the gut microbiota composition in the two groups. Results: Supplementation with Kebirah^®^ prevented the decrease in the Shannon diversity index of gut microbiota, which was instead observed in patients receiving the placebo. In addition, decreases in lymphocyte count and haemoglobin levels were observed only in the placebo group and not in the treated group, which was also characterised by an amelioration of the gut microbial profile, with an enrichment in beneficial bacteria and a preservation of species diversity. Conclusions: Our data suggest that modulating the gut microbiota in acute disease through administration of a specific symbiotic formula could be a useful strategy in the frame of SARS-CoV-2 infections.

## 1. Introduction

Coronavirus disease 2019 (COVID-19), sustained by severe acute respiratory syndrome coronavirus 2 (SARS-CoV-2), is a contagious infection mainly presenting as an acute respiratory disease. It continues to represent a major medical challenge worldwide. In addition, a significant number of infected people experience a sequelae of post-acute long term effects, namely, ongoing, relapsing, or new symptoms which persist for more than 4 weeks after acute COVID-19 infection, a condition referred to as long COVID-19 [1].

A number of factors, including age, sex, genetics, and co-morbidities, are recognized as responsible for different clinical evolutions of COVID-19, ranging from asymptomatic, mild, and moderate to severe or critical [2]. The existence of a gut–lung axis, a bidirectional network in which intestinal bacterial products are able to influence immune responses in the airways, can affect the gut microbiota profile [3], and this has raised the question of whether gut microbiota could represent another risk or prognostic factor for COVID-19. Since the spread of the pandemic, indeed, different studies have described dysbiotic conditions in COVID-19 patients as compared to uninfected individuals [4,5,6], as well as in severely affected subjects with respect to patients with milder disease [2,5,7,8]. As a general finding, an increase in pro-inflammatory and pathogenic and a decrease in beneficial anti-inflammatory and short-chain fatty acid (SCFA)-producing bacteria have been observed in COVID-19, especially in its most serious forms [5,9].

It has been suggested that strategies of microbiota manipulation aimed at shaping a healthier bacterial profile could enhance the host immune system to fight against SARS-CoV-2 infection, thus preventing the infection or ameliorating its clinical outcome [10]. Among these approaches, preliminary studies evaluating probiotics-based interventions as supportive treatment for COVID-19 have revealed some clinical improvements [11,12,13,14]. A recent systematic review evaluated the efficacy of probiotics and prebiotics on COVID-19 and reported a positive effect in terms of a reduction in disease severity, inflammation, and mortality rate [15].

In light of these observations, the current study primarily aimed to assess whether gut microbiota manipulation through the administration of a specific synbiotic formula in addition to standard care would be able to ameliorate the diversity and richness of the microbiota profiles. Secondly, it aimed to improve clinical parameters or alleviate acute or post-acute symptoms in hospitalised COVID-19 patients as compared to their counterparts receiving standard care supplemented with a placebo.

## 2. Methods

### 2.1. Study Design and Participants

The randomized, double-blind, placebo-controlled trial was performed to evaluate the efficacy of nutraceutical supplementation in acute COVID-19 inpatients. The study was approved by the Ethics Committee of the National Institute for Infectious Diseases INMI L. Spallanzani Hospital (approval C.E. number 461-2021), and it was conducted in accordance with the Declaration of Helsinki. The eligible patients provided signed informed consent and were enrolled and randomly assigned to the synbiotic or placebo group. Patients, health professionals, and investigators were blinded to the intervention allocation. Between 18th January 2022 and 21st March 2023, adult COVID-19 patients who were admitted to INMI L. Spallanzani hospital due to COVID-19 symptoms were enrolled in our study. Patients who were first admitted to the intensive care unit (ICU) or needed an enteral nutrition were excluded. All strains and respective data were anonymized prior to inclusion.

### 2.2. Study Protocol

#### 2.2.1. Inclusion Criteria

Participation in this study was proposed to all patients consecutively admitted to the wards of the High Isolation Infectious Diseases Unit of the INMI “Lazzaro Spallanzani”, IRCCS, Rome, who presented the following criteria: (1) willingness to participate in the study by signing the informed consent and (2) positive molecular or antigenic swab for SARS-CoV-2 on admission.

#### 2.2.2. Exclusion Criteria

The following patients were not included in the study: (1) patients who refused to provide consent for the study or who were unable to give their consent; (2) patients with an allergy or intolerance to one of the administered compounds; and (3) pregnant women.

Enrolled patients were randomised to receive either treatment or placebo through a matrix that allowed for random distribution into the two study arms. To mitigate selection bias, a stratified randomization method was applied. Age and disease severity, evaluated as the ratio of partial pressure of arterial oxygen to the fraction of inspired oxygen (PaO_2_/FiO_2_), according to the Berlin definition for acute respiratory distress syndrome (ARDS) [16], were identified as stratification factors to create four strata: Stratum 1 (age < 65; PaO_2_/FiO_2_ > 300 mmHg), Stratum 2 (age ≥ 65; PaO_2_/FiO_2_ > 300 mmHg), Stratum 3 (age < 65; PaO_2_/FiO_2_ ≤ 300 mmHg), and Stratum 4 (age ≥ 65; PaO_2_/FiO_2_ ≤ 300 mmHg).

After consent signature, enrolment, and randomisation, patients received the multi-strain synbiotic formula or a placebo. The capsule was taken orally once a day, on an empty stomach, for a total of 35 days. Rectal swabs were collected at patient admission (baseline) and 14 (T14) and 28 days (T28) after starting the study. Blood examinations at baseline, T14, and T28, including blood count, liver and kidney function, pancreatic enzymes, blood glucose, coagulation, LDH, and C-reactive protein, were analysed as in routine clinical practice.

At 180 days, a questionnaire was administered to patients to explore the presence of symptoms potentially related to the post-acute phase of COVID-19. Symptoms were grouped into four categories: non-specific symptoms, such as fatigue, fever, muscle pain, hair loss, blurred vision, rash, and/or cardio-respiratory issues (palpitations, dyspnoea, chest pain, cough, rhinorrhoea, sore throat); gastro-intestinal symptoms (nausea, lack of appetite, intestinal disorders, diarrhoea, ageusia, anosmia); and neurological/cognitive symptoms (headache, anxiety, dizziness, difficulty concentrating, insomnia, paresthesias). A single patient could complain of symptoms in one or more categories at the same time.

### 2.3. Study Products

The multi-strain synbiotic formula (Kebirah^®^) was administered in a capsule containing *Bifidobacterium breve* SGB01 (5 × 10^−9^ CFU), *Bifidobacterium bifidum* SGB02 (5 × 10^−9^ CFU), *Lactobacillus kefiri* SGL13 (7.5 × 10^−9^ CFU), *Lactobacillus rhamnosus* SGL06 (7.5 × 10^−9^ CFU), and *Lactobacillus salivarius* SGL03 (7.5 × 10^−9^ CFU), fructooligosaccharides and galactooligosaccharides (250 mg) as prebiotic agents, quercetin (50 mg), and calcium butyrate (50 mg). In the control group, placebo capsules were administered once daily to the participants. They were indistinguishable in appearance, weight, and packaging as compared to synbiotic capsules, and they contained maltodextrin and silicon dioxide as anticaking agents. They were both provided by NTP srl (Castelnuovo Vomano, Teramo, Italy).

### 2.4. Laboratory and 16S rRNA Gene Sequencing

DNA samples isolated from patients’ faecal swabs were obtained with a Nimbus automatic extractor (Seegene Technologies, Seoul, Republic of Korea) starting from 500 µL of sample. This was performed following the manufacturer’s protocol after treatment with 500 µL of Lysis Buffer ATL (QIAGEN, Hilden, Germany) at 56 °C for 10 min with 20 µL Proteinase K (Darmstadt, Germany). Amplicon sequencing was performed according to the 16S Metagenomics Sequencing Workflow provided by Illumina. Briefly, the hypervariable region V3–V4 of the bacterial 16S rRNA gene was amplified using universal primers selected by Klindworth [17]. Libraries were then barcoded using the dual-index system Nextera XT Index Kit (Illumina, San Diego, CA, USA), and they were then pooled equimolarly to perform multiplex paired-end sequencing (2 × 300 cycles) on a MiSeq device (Illumina). All the other parameters analysed in the trials were obtained from the routine laboratory tests.

### 2.5. Bioinformatics Analysis

De-multiplexed FASTQ files containing raw sequencing data, deposited in ArrayExpress under the accession code E-MTAB-14041, were analysed using the 16S Metagenomics GAIA 2.0 software (Sequentia Biotech, Barcelona, Spain, 2019). Read pairs underwent quality checks (i.e., trimming, clipping and adapter removal) based on FastQC and BBDuk. Quality-controlled sequences were mapped against the NCBI 16S reference database to obtain taxonomic assignments. Within-sample diversity, expressed by the Shannon evenness index and the Chao1 richness index, was also computed by the software.

### 2.6. Statistical Analysis

The clinical characteristics of the patients were reported as means and standard deviations, medians and interquartile ranges, and frequencies (both absolute and percentages) for continuous and categorical variables, respectively. The assumption of normality for the distribution of continuous variables was evaluated using the Shapiro–Wilk test. Comparisons of clinical characteristics between the two treatment arms were performed using a two-sample *t*-test or Mann–Whitney U test as appropriate, or the Chi-Square test or Fisher’s exact test for continuous and categorical variables, respectively. The magnitude of these differences was also assessed by the standardized mean difference (SMD). This measure, which can also be applied to proportions, quantifies the overall difference in terms of “effect size” (i.e., indicating clinical relevance). As a general guideline, SMD values of 0.20, 0.50, and 0.80 are considered to denote small, medium, and large effect sizes, respectively [18].

The assessment of gut microbiota alpha-diversity (i.e., richness and evenness indices) over time, as well as haematochemical parameters, was carried out using repeated-measures ANOVA models. Comparisons within and between groups were performed using the appropriate statistical contrasts defined in the model, imposing an unstructured covariance matrix on the residuals. Each model included the following independent variables: group (probiotic vs. placebo), time (as a categorical variable: T0 vs. T14 vs. T28), and the group-by-time interaction term. The *p*-value from the interaction term (type III test) evaluated whether the change over time in the outcome variable differed between the two groups. Due to their skewed distribution, the values of haematochemical parameters were log-transformed prior to statistical analysis. Two-sided *p*-values < 0.05 denoted statistical significance. All statistical analyses were performed using SAS software (version 9.4, SAS Institute Inc., Cary, NC, USA).

As for microbiota composition, the differential analysis was performed using DESeq2 analysis (Version 1.44.0). Results were considered significant when the FDR-adjusted *p* value was <0.05.

### 2.7. Sample Size Calculation

To determine the required number of patients for each arm of the study, it was hypothesized that the probiotic would maintain the composition (both richness and evenness) of the gut microbiota at 14 days post-treatment initiation. Conversely, it was expected that patients in the placebo arm would experience a significant disruption (or loss) of gut flora due to the acute phase of COVID-19. After 14 days, both groups were expected to return to baseline in terms of microbiota composition. Given the randomization, a comparison of the gut microbiota composition (richness and evenness) after 14 days between the two independent treatment groups would suffice. Formally, it was determined that a total of 52 randomized (1:1) patients would provide 80% power to detect an SMD of 0.80 in the richness or evenness indices between the two groups at a significance level (alpha) of 0.05 using a two-sided, two-sample *t*-test.

## 3. Results

### 3.1. Study Population and Safety 

**Figure 1:**
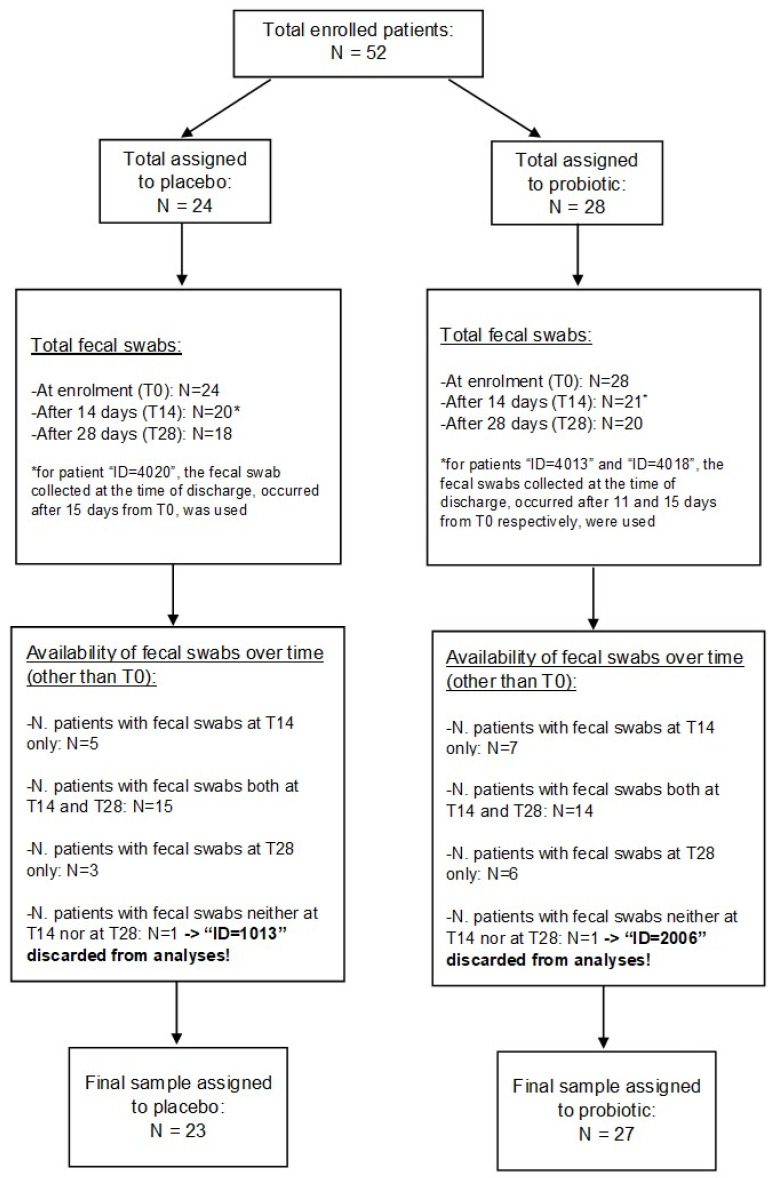
CONSORT Flow diagram of patient disposition.

A total of 52 patients were enrolled in our study. Of these, 24 were allocated to the placebo group and the remaining 28 to the probiotic group. One patient from each group was excluded from the statistical analyses due to the absence of faecal samples at the specific time points (i.e., at least one measurement after T0 was required). Figure 1 shows the flow diagram of the study design.

The demographic and clinical characteristics of the study sample at enrolment are reported in Table 1. No statistically significant differences were observed between the two groups, indicating the success of the randomization scheme. No serious adverse events were reported among the study subjects.

### 3.2. Analysis of Haematochemical Parameters in the Synbiontic and Placebo Groups over Time 

The median value and interquartile range of each haematochemical parameter at baseline and after 14 and 28 days are reported in Table 2. It was shown that synbiotic formula supplementation prevented decreases in lymphocyte count and haemoglobin levels, but this was observed only in the placebo group and not in the treated group. LDH levels significantly decreased in the placebo group at both T14 (*p* = 0.015) and T28 (*p* = 0.034) compared to T0. This was not observed in the probiotic group, although all values were within the normal range for both groups. Conversely, CRP levels significantly decreased at T14 and T28 in both groups compared to T0 (all *p* < 0.001).

Other acute-phase reactants, including ferritin and fibrinogen, displayed changes in both the control and treated groups. Ferritin levels initially increased at T14 and then significantly reduced at T28 within each group, although this decrease (T28 vs. T14) was statistically significant only in the placebo group (*p* = 0.032). It is important to note that ferritin levels could have been influenced by the presence of other co-morbid conditions and/or confounders, and that these levels remained within the normal range. Although the fibrinogen levels were found to be increased in patients requiring hospitalization for COVID-19 [19], a significant decrease was observed at T14 in the placebo group (*p* = 0.014). However, it should be noted that, in the frame of COVID-19, fibrinogen plays a key role in protecting the host, as was elegantly suggested by Tachil [20]. This indicates that probiotics administration also exerts a protective role, at least in part due to sustained fibrinogen levels over time. 

**Figure 2:**
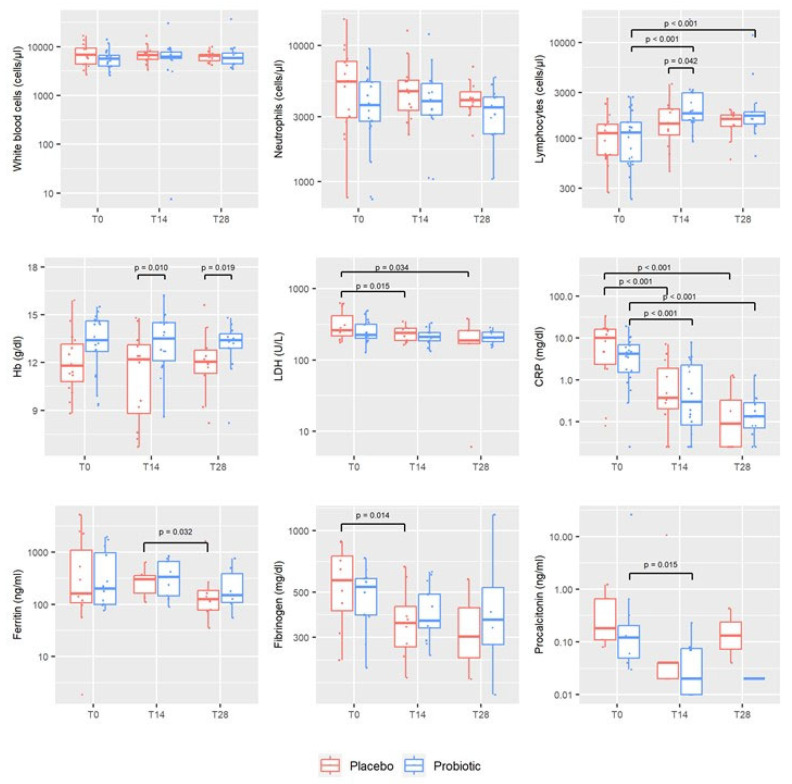
Boxplots of haematochemical parameters assessed in patients with probiotic and placebo administration at enrolment (T0) and after 14 and 28 days (T14 and T28), respectively.

As for procalcitonin, a promising prognostic biomarker of COVID-19 progression whose overexpression can strongly predict poor outcomes in COVID-19 patients [21,22,23], it was found to be significantly decreased at T14 in the probiotic group (*p* = 0.015). The distribution of haematochemical parameters at T0 and over time is shown in Figure 2 using boxplots.

### 3.3. Assessment of Patients’ Health Statuses via Telephone Questionnaire at 6 Months after Enrolment

At 6 months after enrolment, a questionnaire was administered to the patients in order to assess their health statuses (Table 3). Although no statistically significant differences were observed between the two groups in terms of disease-related symptoms, it is worth mentioning that, despite the limited number of observations, fewer patients in the probiotics group exhibited moderate to severe neurological/neurocognitive symptoms (9.1%) compared to those in the placebo group (35%), denoting a medium effect size (SMD = 0.66). Conversely, only 90.9% of patients belonging to the probiotics group reported moderate/severe neurological/neurocognitive symptoms, as compared to 65% of patients within the placebo group. A similar trend was also observed for digestive symptoms.

### 3.4. Comparison of Gut Microbiota Composition in Probiotic Versus Placebo Groups over Time

**Figure 3:**
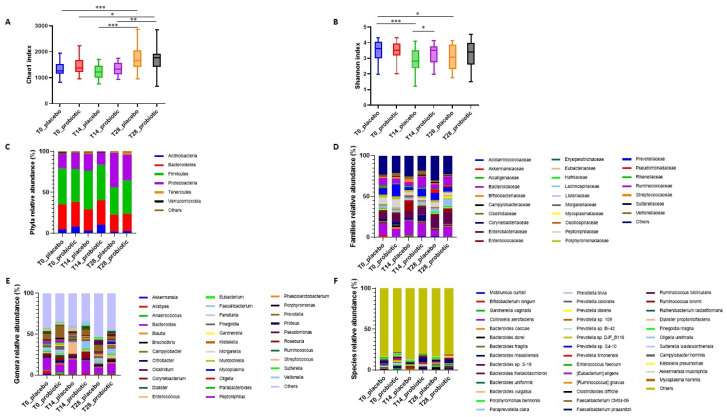
Alpha-diversity indices and taxonomic composition of gut microbiota. Boxplots representing species-level richness expressed by Chao1 index (**A**) and evenness expressed by Shannon Index (**B**) in the two cohorts at different time points. Significance levels were as follows: * *p* ≤ 0.05, ** *p* ≤ 0.01, *** *p* ≤ 0.001. Stacked barplots showing the mean relative abundance of gut bacteria at the phylum (**C**), family (**D**), genus (**E**), and species (**F**) level.

The 16S rRNA sequencing analysis of gut microbiota composition from rectal swabs of patients at T0, T14, and T28 produced an average of 191,139.9 quality-controlled read pairs per sample. When the alpha-diversity metrics were analysed (Table 4, Figure 3A,B), no statistically significant differences were found in the species richness (i.e., the Chao1 index) at T14 vs. T0 in either the placebo or probiotic group. However, a significant increase was evidenced at T28 vs. T0 and at T28 vs. T14 within both groups. Regarding the species evenness, expressed by the Shannon index, a statistically significant decrease was observed at T14 vs. T0 in the placebo group (*p* = 0.001), but not in the probiotics one. Consequently, a statistically significant difference in this index mean was observed between the two groups at T14 (*p* = 0.029), achieving a medium effect size (SMD = 0.74). Generally, the Shannon index did not show significant variation over time in the probiotic group, suggesting that the probiotic preserved the gut species evenness during the acute phase of COVID-19.

As for the composition, the gut microbial profiles at the phylum, family, genus, and species levels are represented in Figure 3C–F. Since some differences in haematochemical parameters upon probiotics administration as compared to placebo were observed 14 days after enrolment, we decided to focus on comparing the gut microbiota composition between the two groups at T14.

## 4. Discussion

A conspicuous body of literature has shown gut dysbiosis to be associated with COVID-19, the severity of its symptoms, and its recovery [2,4,5,6,7,8] due to the existence of the so-called gut–lung axis and to the ability of intestinal bacterial metabolites to modulate the host immune system. Therefore, the manipulation of gut microbiota through the administration of probiotics for restoring a favourable microbiota could represent a valid supportive tool for boosting immunity and managing the disease.

In our study, supplementation with Kebirah^®^ modified the haematochemical parameters. Lymphopenia is common in viral infections [24]. In particular, this finding has been associated with severe COVID-19, and it has been suggested to be useful in predicting the severity of clinical outcomes [25]. We observed that probiotics supplementation prevented the decrease in lymphocyte count, suggesting a protective role of probiotics during SARS-CoV-2 infection. Similarly, decreased haemoglobin levels have been associated with worse evolution [26]. We did not observe decreased haemoglobin levels in the probiotics group, but only in the placebo group. A further beneficial effect in probiotics-receiving patients was the decrease in calcitonin levels after 14 days of treatment. Besides being a biomarker for bacterial sepsis, an increase in procalcitonin serum levels has been found in COVID-19 patients as well, as a consequence of the massive cytokine production characterizing the most severe forms of the disease [21,22,23]. Moreover, concerning post-acute COVID-19 manifestations, patients belonging to the probiotics group displayed fewer neurological/neurocognitive symptoms than placebo group, tending towards significance without reaching it (0.062), with a medium effect size (0.658). This result was not surprising, as with small sample sizes, only large or very large effect sizes can be detected as statistically significant. However, a meta-analysis that combines all the studies can demonstrate evidence for a treatment benefit [27,28,29]. These beneficial effects on COVID-19 symptoms were mediated by the microbiota-modulating effect of the administered synbiotic formula, and they are in agreement with previous studies demonstrating the efficacy of probiotic treatments in the management of the disease [11,12,13,14]. The well-established existence of a gut–lung axis, in which gut bacteria and their metabolites enter the systemic circulation and then reach the lungs, where they shape immune responses, provides rationale for the efficacy of strategies of microbiota manipulation in the treatment of COVID-19 [3,30], a disease in which the inflammatory component plays a pivotal role. This also applies to the post-acute phase of the disease, since there is evidence that a persistently dysregulated immune response underlies the prolonged symptoms characterizing long COVID-19 [31,32].

In the current study, supplementation with Kebirah^®^ had the effect of preventing the decrease in the Shannon diversity index, which was instead observed in patients receiving a placebo. Previous studies have reported a reduction in alpha-diversity in COVID-19 patients as compared to uninfected controls [33,34], as well as in more severe forms of the disease compared to the milder ones [7].

In differential abundance testing using DESeq2, among the significant shifts between the two cohorts after 14 days of treatment, an increase in the phylum of *Actinobacteria* was observed in patients treated with probiotics as compared to those receiving placebo. This result was surprising, since we have previously described a higher *Actinobacteria* abundance in patients with severe COVID-19 symptoms with respect to patients with mild disease [2]. It should be noted, however, that in our previous report, the major contribution to *Actinobacteria* increase was given by the family of *Corynebacteriaceae*, whose members have been described to cause respiratory tract infections [2]. On the contrary, in the current study, the family of *Bifidobacteriaceae* was the main one responsible for *Actinobacteria* enrichment in probiotic-treated subjects, and it was accompanied by a significant increase in *Bifidobacterium* and *Collinsella* (both belonging to *Actinobacteria*). Interestingly, these genera were previously found to be under-represented in COVID-19 patients with moderate/severe disease as compared to those with mild disease [7]. In another study, moreover, *Collinsella* was found to predict low mortality rates in COVID-19 patients, presumably through the production of ursodeoxycholate, which has anti-inflammatory and anti-oxidant functions [35].

Another feature of the gut microbiota profile described in patients with moderate/severe COVID-19 was the lower abundance of butyrate-producing bacteria, such as *Roseburia*, *Faecalibacterium,* and *Eubacterium* [7,8]. Interestingly, in our study, 14 days of probiotics supplementation resulted in a statistically significant enrichment in all the aforementioned genera and species therein (e.g., *Roseburia faecis*, *Roseburia hominis*, *Roseburia inulinovorans*, *Eubacterium eligens*), as well as in other butyrate producers, including *Anaerostipes hadrus*, *Ruminococcus faecis*, *Ruminococcus lactaris*, *Butyricicoccus faecihominis*, *Lachnoclostridium phocaeense*, and *Clostridium* sp. BPY5. The majority of butyrate produced by bacteria is used by colonocytes as an energy source, but butyrate is also known to exert its beneficial effects at a systemic level due to its immunomodulatory and anti-inflammatory functions [36,37]. Moreover, a reduction in anti-inflammatory SCFAs, including butyrate, has been related to psychiatric symptoms in the context of COVID-19, suggesting an interconnection between long COVID-19, psychiatric symptoms, and cognitive impairment [38].

Noteworthily, our study also revealed a significant decrease in the relative abundance of *Enterococcus faecium* in the interventional arm with respect to the placebo arm. This opportunistic pathogen has been described to be among the main pathogens responsible for co-infections in critically ill COVID-19 patients [39], has been found to be enriched in the guts of subjects with severe COVID-19 [40], and has been correlated with a poor prognosis [41].

The current study is a monocentric clinical study, which could represent a limitation. Moreover, further in vitro and in vivo study models should be employed in order to obtain more mechanistic insights and to more deeply understand the observed phenomena.

To summarize, an overall amelioration in the gut microbial profile, with an enrichment in beneficial bacteria and a preservation of species diversity, was recorded in probiotics-treated patients as compared to those taking a placebo. These favourable changes in gut microbiota were accompanied by significant amelioration of some clinical markers which were altered during the acute phase of the disease, and by a tendency towards improvement in other long COVID-19 symptoms, suggesting that modulating the gut microbiota through administration of specific probiotics could be a useful strategy in the frame of SARS-CoV-2 infections.

## Figures and Tables

**Table 1:** Demographic and clinical characteristics of the study sample at enrolment (i.e., first faecal swab collection).

		Total (N = 50)	Placebo (N = 23)	Probiotic (N = 27)	SMD	*p*-Value	% of Subjects with Missing Info
Age (years)	Mean ± SD	67.8 ± 12.9	67.9 ± 11.8	67.7 ± 14.0	0.010	0.972 ^1^	0%
	Median [IQR]	70.0 [60.0–77.0]	67.0 [60.0–79.0]	71.0 [60.0–75.0]			
	Min–Max	32.0–89.0	46.0–87.0	32.0–89.0			
Sex—N (%)	Female	21 (42.0)	11 (47.8)	10 (37.0)	0.220	0.441 ^3^	0%
	Male	29 (58.0)	12 (52.2)	17 (63.0)			
WHO-CPS (scale)	Mean ± SD	4.7 ± 0.8	4.7 ± 1.1	4.8 ± 0.6	0.146	0.837 ^2^	0%
	Median [IQR]	5.0 [4.0–5.0]	5.0 [4.0–5.0]	5.0 [4.0–5.0]			
	Min–Max	1.0–6.0	1.0–6.0	4.0–6.0			
State at hospitalization—N (%)	Asymptomatic	1 (2.0)	1 (4.3)	0 (0.0)	0.302	0.283 ^4^	0%
*(based on WHO-CPS)*	Moderate	43 (86.0)	18 (78.3)	25 (92.6)	0.415		
	Severe	6 (12.0)	4 (17.4)	2 (7.4)	0.306		
SARS-CoV-2 vaccine (type)—N (%)	No (unvaccinated)	7 (14.0)	2 (8.7)	5 (18.5)	0.289	0.388 ^4^	0%
	Astrazeneca	2 (4.0)	0 (0.0)	2 (7.4)	0.400		
	Moderna	1 (2.0)	1 (4.3)	0 (0.0)	0.302		
	Pfizer	32 (64.0)	17 (73.9)	15 (55.6)	0.392		
	Unknown	8 (16.0)	3 (13.0)	5 (18.5)	0.151		
Number of vaccine doses	Mean ± SD	2.6 ± 1.2	2.8 ± 1.0	2.4 ± 1.3	0.317	0.262 ^2^	0%
	Median [IQR]	3.0 [3.0–3.0]	3.0 [3.0–3.0]	3.0 [2.0–3.0]			
	Min–Max	0.0–4.0	0.0–4.0	0.0–4.0			
Comorbidies—N (%)	Yes	46 (92.0)	22 (95.7)	24 (88.9)	0.255	0.614 ^4^	0%
Diabetes—N (%)	Yes	12 (24.0)	8 (34.8)	4 (14.8)	0.475	0.099 ^3^	0%
Cardiovascular disease—N (%)	Yes	13 (26.0)	6 (26.1)	7 (25.9)	0.004	0.990 ^3^	0%
Ipertension—N (%)	Yes	26 (52.0)	11 (47.8)	15 (55.6)	0.155	0.586 ^3^	0%
Obesity—N (%)	Yes	7 (14.6)	4 (18.2)	3 (11.5)	0.188	0.687 ^4^	2%
Neoplasm—N (%)	Yes	11 (22.0)	5 (21.7)	6 (22.2)	0.012	0.967 ^3^	0%
COPD—N (%)	Yes	11 (22.0)	5 (21.7)	6 (22.2)	0.012	0.967 ^3^	0%
Immunodeficiency—N (%)	Yes	12 (24.0)	6 (26.1)	6 (22.2)	0.090	0.750 ^3^	0%
Renal failure—N (%)	Yes	4 (8.0)	4 (17.4)	0 (0.0)	0.649	0.038 ^4^	0%
Other comorbidies—N (%)	Yes	7 (14.0)	1 (4.3)	6 (22.2)	0.546	0.107 ^4^	0%
Gastrointestinal diseases—N (%)	Yes	1 (2.0)	1 (4.3)	0 (0.0)	0.302	0.460 ^4^	0%
Diarrhoea—N (%)	Yes	1 (2.0)	1 (4.3)	0 (0.0)	0.302	0.460 ^4^	0%
Sotrovimab during hospitalization—N (%)	Yes	9 (20.0)	5 (25.0)	4 (16.0)	0.224	0.482 ^4^	5%
Pump inhibitors—N (%)	Yes	36 (73.5)	17 (73.9)	19 (73.1)	0.019	0.947 ^3^	1%
Antibiotics—N (%)	Yes	30 (60.0)	15 (65.2)	15 (55.6)	0.199	0.487 ^3^	0%
Steroids—N (%)	Yes	27 (54.0)	12 (52.2)	15 (55.6)	0.068	0.811 ^3^	0%
Remdesivir—N (%)	Yes	40 (80.0)	18 (78.3)	22 (81.5)	0.080	1.000 ^4^	0%
Immunomodulants—N (%)	No	41 (91.1)	17 (85.0)	24 (96.0)	0.382	0.436 ^4^	5%
	Anakinra	2 (4.4)	1 (5.0)	1 (4.0)	0.048		
	Baricitinib	2 (4.4)	2 (10.0)	0 (0.0)	0.471		
Pneumonia—N (%)	Si	44 (88.0)	21 (91.3)	23 (85.2)	0.191	0.674 ^4^	0%
Oxygen therapy—N (%)	Si	39 (78.0)	18 (78.3)	21 (77.8)	0.012	0.967 ^3^	0%
Length of stay (days)	Mean ± SD	16.5 ± 9.4	16.8 ± 11.3	16.3 ± 7.6	0.054	0.654 ^2^	0%
	Median [IQR]	15.5 [10.0–22.0]	15.0 [8.0–22.0]	17.0 [10.0–22.0]			
	Min–Max	4.0–53.0	5.0–53.0	4.0–33.0			
Clinical outcome—N (%)	Discharged	48 (96.0)	22 (95.7)	26 (96.3)	0.033	1.000 ^4^	0%
	Dead (due to COVID)	2 (4.0)	1 (4.3)	1 (3.7)			

^1^ *p*-value from two-sample *t*-test; ^2^ *p*-value from Mann–Whitney U test; ^3^ *p*-value from Chi-Square test; ^4^ *p*-value from Fisher’s exact test. Abbreviations: SD: standard deviation; IQR: interquartile range (i.e., first–third quartiles); SMD: standardized mean difference. Notes: SMD is defined as the difference between the means (or proportions) of two samples divided by the standard deviation of this difference. This measure of effect size, also known as Cohen’s d, represents the mean difference in terms of its homogeneity. As a general guideline, SMD values of 0.20, 0.50, and 0.80 are considered to denote small, medium, and large effect sizes, respectively.

**Table 2:** Analysis of haematochemical parameters assessed in patients with probiotic and placebo administration (groups) at enrolment (T0) and after 14 and 28 days (T14 and T28), respectively.

						Within-Group Comparisons			
	Group	Statistic	T0	T14	T28	T14 vs. T0	T28 vs. T0	T28 vs. T14	Test for Differential Trend *
White Blood Cells (cells/µL)	Placebo	N.obs	16	12	11	0.984	0.567	0.771	0.424
		Median [IQR]	6890.0 [4410.0–9825.0]	6810.0 [5405.0–8010.0]	6490.0 [4660.0–7170.0]				
	Probiotic	N.obs	21	17	13	0.574	0.347	0.292	
		Median [IQR]	5630.0 [4040.0–6590.0]	6240.0 [5760.0–7780.0]	5870.0 [4470.0–7390.0]				
	**Between-group comparisons**	--	0.242	0.436	0.807	--	--	--	
Neutrophils (cells/µL)	Placebo	N.obs	16	12	11	0.872	0.250	0.180	0.869
		Median [IQR]	5450.0 [2885.0–7770.0]	4629.5 [3200.0–5665.0]	3970.0 [3450.0–5030.0]				
	Probiotic	N.obs	21	17	13	0.334	0.520	0.078	
		Median [IQR]	3650.0 [2770.0–5420.0]	3900.0 [3080.0–5270.0]	3500.0 [2240.0–4180.0]				
	**Between-group comparisons**	--	0.168	0.309	0.164	--	--	--	
Lymphocytes (cells/µL)	Placebo	N.obs	16	12	11	0.076	0.016	0.773	0.248
		Median [IQR]	1125.0 [655.0–1420.0]	1420.0 [1005.0–2020.0]	1580.0 [1320.0–1790.0]				
	Probiotic	N.obs	21	17	13	<0.001	<0.001	0.263	
		Median [IQR]	1140.0 [570.0–1460.0]	1810.0 [1530.0–2970.0]	1710.0 [1410.0–1870.0]				
	**Between-group comparisons**	--	0.972	0.042	0.161	--	--	--	
Hb (g/dL)	Placebo	N.obs	15	12	10	0.054	0.132	0.604	0.159
		Median [IQR]	11.8 [10.4–13.4]	12.2 [8.4–13.2]	12.1 [11.2–12.9]				
	Probiotic	N.obs	21	17	13	0.426	0.798	0.548	
		Median [IQR]	13.4 [12.7–14.6]	13.5 [12.1–14.5]	13.4 [12.9–13.8]				
	**Between-group comparisons**	--	0.087	0.010	0.019	--	--	--	
LDH (U/L)	Placebo	N.obs	11	8	5	0.015	0.034	0.120	0.456
		Median [IQR]	259.0 [193.0–568.0]	239.5 [183.5–279.0]	186.0 [170.0–257.0]				
	Probiotic	N.obs	16	13	7	0.176	0.249	0.403	
		Median [IQR]	224.5 [197.0–322.5]	209.0 [184.0–241.0]	206.0 [163.0–251.0]				
	**Between-group comparisons**	--	0.201	0.821	0.455	--	--	--	
CRP (mg/dL)	Placebo	N.obs	14	11	9	<0.001	<0.001	0.136	0.680
		Median [IQR]	10.3 [1.8–16.5]	0.4 [0.2–3.0]	0.1 [0.0–0.3]				
	Probiotic	N.obs	20	16	12	<0.001	<0.001	0.102	
		Median [IQR]	4.2 [1.4–7.3]	0.3 [0.1–2.6]	0.1 [0.1–0.3]				
	**Between-group comparisons**	--	0.368	0.907	0.983	--	--	--	
Ferritin (ng/mL)	Placebo	N.obs	11	6	8	0.784	0.362	0.032	0.619
		Median [IQR]	160.0 [96.0–2248.0]	302.5 [134.0–372.0]	126.5 [76.0–214.5]				
	Probiotic	N.obs	12	9	6	0.783	0.396	0.198	
		Median [IQR]	200.0 [99.5–1085.0]	332.0 [146.0–660.0]	152.0 [104.0–495.0]				
	**Between-group comparisons**	--	0.777	0.919	0.557	--	--	--	
Fibrinogen (mg/dL)	Placebo	N.obs	8	8	3	0.014	0.264	0.761	0.433
		Median [IQR]	576.5 [377.5–796.0]	352.5 [260.5–487.0]	303.0 [187.0–578.0]				
	Probiotic	N.obs	13	13	4	0.212	0.868	0.643	
		Median [IQR]	531.0 [386.0–583.0]	362.0 [334.0–489.0]	367.0 [245.5–800.0]				
	**Between-group comparisons**	--	0.495	0.450	0.699	--	--	--	
Procalcitonin (ng/mL)	Placebo	N.obs	5	5	2	0.269	NA	NA	0.566
		Median [IQR]	0.2 [0.1–0.7]	0.0 [0.0–0.0]	0.2 [0.0–0.4]				
	Probiotic	N.obs	11	11	1	0.015	NA	NA	
		Median [IQR]	0.1 [0.0–0.3]	0.0 [0.0–0.1]	0.0 [0.0–0.0]				
	**Between-group comparisons**	--	0.646	0.262	NA	--	--	--	

* is the *p*-value from time-by-group interaction (type III test) term, which assesses whether the change over time of the outcome variable is differential between the two groups. Abbreviations: N.obs: Number of patients with available data; IQR: interquartile range (i.e., first–third quartiles); NA: not available (computable) due to the large amount of missing data. Notes: Only patients with at least one measurement after T0 were considered in the analysis. Because the analyses were performed on log-transformed variables, only median and IQR were reported as descriptive statistics.

**Table 3:** Health statuses of patients assessed by telephone questionnaire at 6 months after enrolment.

	Category	Total (N = 50)	Placebo (N = 23)	Probiotic (N = 27)	SMD	*p*-Value ^#^	% of Subjects with Missing Info
Breathlessness—N (%)	No/Mild	37 (88.1)	19 (95.0)	18 (81.8)	0.421	0.346	16%
	Moderate/Severe	5 (11.9)	1 (5.0)	4 (18.2)			
Fatigue—N (%)	No/Mild	30 (71.4)	15 (75.0)	15 (68.2)	0.152	0.738	16%
	Moderate/Severe	12 (28.6)	5 (25.0)	7 (31.8)			
Muscle pain—N (%)	No/Mild	34 (81.0)	17 (85.0)	17 (77.3)	0.198	0.700	16%
	Moderate/Severe	8 (19.0)	3 (15.0)	5 (22.7)			
Chest pain—N (%)	No/Mild	40 (95.2)	19 (95.0)	21 (95.5)	0.021	1.000	16%
	Moderate/Severe	2 (4.8)	1 (5.0)	1 (4.5)			
Headache —N (%)	No/Mild	41 (97.6)	19 (95.0)	22 (100.0)	0.324	0.476	16%
	Moderate/Severe	1 (2.4)	1 (5.0)	0 (0.0)			
Cough—N (%)	No/Mild	41 (97.6)	19 (95.0)	22 (100.0)	0.324	0.476	16%
	Moderate/Severe	1 (2.4)	1 (5.0)	0 (0.0)			
Fever—N (%)	No/Mild	42 (100.0)	20 (100.0)	22 (100.0)	<0.001	1.000	16%
	Moderate/Severe	0 (0.0)	0 (0.0)	0 (0.0)			
Rhinorrhoea —N (%)	No/Mild	41 (97.6)	19 (95.0)	22 (100.0)	0.324	0.476	16%
	Moderate/Severe	1 (2.4)	1 (5.0)	0 (0.0)			
Sore throat—N (%)	No/Mild	42 (100.0)	20 (100.0)	22 (100.0)	<0.001	1.000	16%
	Moderate/Severe	0 (0.0)	0 (0.0)	0 (0.0)			
Nausea—N (%)	No/Mild	40 (97.6)	18 (94.7)	22 (100.0)	0.333	0.463	18%
	Moderate/Severe	1 (2.4)	1 (5.3)	0 (0.0)			
Lack of appetite —N (%)	No/Mild	39 (92.9)	19 (95.0)	20 (90.9)	0.160	1.000	16%
	Moderate/Severe	3 (7.1)	1 (5.0)	2 (9.1)			
Ageusia—N (%)	No/Mild	38 (97.4)	18 (94.7)	20 (100.0)	0.333	0.487	22%
	Moderate/Severe	1 (2.6)	1 (5.3)	0 (0.0)			
Anosmia—N (%)	No/Mild	42 (100.0)	20 (100.0)	22 (100.0)	<0.001	1.000	16%
	Moderate/Severe	0 (0.0)	0 (0.0)	0 (0.0)			
Anxiety —N (%)	No/Mild	38 (90.5)	17 (85.0)	21 (95.5)	0.358	0.333	16%
	Moderate/Severe	4 (9.5)	3 (15.0)	1 (4.5)			
Dizziness—N (%)	No/Mild	40 (97.6)	18 (94.7)	22 (100.0)	0.333	0.463	18%
	Moderate/Severe	1 (2.4)	1 (5.3)	0 (0.0)			
Concentration difficulties—N (%)	No/Mild	38 (90.5)	18 (90.0)	20 (90.9)	0.031	1.000	16%
	Moderate/Severe	4 (9.5)	2 (10.0)	2 (9.1)			
Insomnia—N (%)	No/Mild	34 (87.2)	15 (78.9)	19 (95.0)	0.491	0.182	22%
	Moderate/Severe	5 (12.8)	4 (21.1)	1 (5.0)			
Hair loss—N (%)	No/Mild	38 (92.7)	17 (89.5)	21 (95.5)	0.228	0.588	18%
	Moderate/Severe	3 (7.3)	2 (10.5)	1 (4.5)			
Palpitations—N (%)	No/Mild	39 (97.5)	18 (100.0)	21 (95.5)	0.309	1.000	20%
	Moderate/Severe	1 (2.5)	0 (0.0)	1 (4.5)			
Intestinal disorders—N (%)	No/Mild	35 (87.5)	14 (77.8)	21 (95.5)	0.538	0.155	20%
	Moderate/Severe	5 (12.5)	4 (22.2)	1 (4.5)			
Blurred vision—N (%)	No/Mild	37 (90.2)	17 (89.5)	20 (90.9)	0.048	1.000	18%
	Moderate/Severe	4 (9.8)	2 (10.5)	2 (9.1)			
Paresthesias —N (%)	No/Mild	39 (95.1)	17 (89.5)	22 (100.0)	0.485	0.209	18%
	Moderate/Severe	2 (4.9)	2 (10.5)	0 (0.0)			
Rash—N (%)	No/Mild	36 (97.3)	17 (100.0)	19 (95.0)	0.324	1.000	26%
	Moderate/Severe	1 (2.7)	0 (0.0)	1 (5.0)			
Cardio-respiratory symptoms (Group 1)	No/Mild	35 (83.3)	17 (85.0)	18 (81.8)	0.086	1.000	16%
	Moderate/Severe	7 (16.7)	3 (15.0)	4 (18.2)			
Digestive symptoms (Group 2)	No/Mild	34 (81.0)	14 (70.0)	20 (90.9)	0.547	0.123	16%
	Moderate/Severe	8 (19.0)	6 (30.0)	2 (9.1)			
Neurological/ neurocognitive symptoms (Group 3)	No/Mild	33 (78.6)	13 (65.0)	20 (90.9)	0.658	0.062	16%
	Moderate/Severe	9 (21.4)	7 (35.0)	2 (9.1)			
Systemic symptoms (Group 4)	No/Mild	24 (57.1)	12 (60.0)	12 (54.5)	0.110	0.764	16%
	Moderate/Severe	18 (42.9)	8 (40.0)	10 (45.5)			

^#^ *p*-values from Fisher’s exact test; Abbreviations: SMD: standardized mean difference. Notes: SMD is defined as the difference between the means (or proportions) of two samples divided by the standard deviation of this difference. This measure of effect size, also known as Cohen’s d, represents the mean difference in terms of its homogeneity. As a general guideline, SMD values of 0.20, 0.50, and 0.80 are considered to denote small, medium, and large effect sizes, respectively.

**Table 4:** Analysis of Chao1 and Shannon indices assessed in patients with probiotic and placebo administration (groups) at enrolment (T0) and after 14 and 28 days (T14 and T28), respectively.

						Within-Group Comparisons			
	Group	Statistic	T0	T14	T28	T14 vs. T0	T28 vs. T0	T28 vs. T14	Test for Differential Trend *
Chao1	Placebo	N.obs	22	12	17	0.404	0.001	0.001	0.002
		Mean ± SD	1332.7 ± 279.6	1216.9 ± 289.4	1761.9 ± 493.2				
		Median [IQR]	1257.6 [1154.8–1530.9]	1231.8 [1004.2–1457.4]	1650.5 [1418.7–2035.3]				
	Probiotic	N.obs	24	17	18	0.228	0.023	0.008	
		Mean ± SD	1443.1 ± 330.7	1352.8 ± 254.8	1753.4 ± 548.1				
		Median [IQR]	1404.9 [1220.4–1576.1]	1320.6 [1151.2–1551.0]	1769.4 [1436.2–1861.8]				
	**Between-group comparisons**	--	0.230	0.410	0.759	--	--	--	
Shannon	Placebo	N.obs	22	12	17	<0.001	0.030	0.232	0.004
		Mean ± SD	3.5 ± 0.6	2.7 ± 0.9	3.0 ± 0.8				
		Median [IQR]	3.7 [3.0–4.0]	2.7 [2.4–3.5]	3.1 [2.4–3.7]				
	Probiotic	N.obs	24	17	18	0.288	0.169	0.597	
		Mean ± SD	3.5 ± 0.5	3.3 ± 0.6	3.2 ± 0.9				
		Median [IQR]	3.6 [3.2–4.0]	3.5 [2.8–3.7]	3.4 [2.7–3.9]				
	**Between-group comparisons**	--	0.947	0.029	0.522	--	--	--	

* is the *p*-value from the time-by-group interaction (type III test) term, which assesses whether the change over time in the outcome variable is differential between the two groups. Abbreviations: N.obs: Number of patients with available data; SD: standard deviation; IQR: interquartile range (i.e., first-third quartiles). Note: Only patients with at least one measurement after T0 were considered in the analysis.

## Data Availability

Raw sequencing data, deposited in ArrayExpress under the accession code E-MTAB-14041.
